# Efficacy of Traditional Chinese Medicine Regimen Jian Pi Qu Shi Formula for Refractory Patients with Idiopathic Membranous Nephropathy: A Retrospective Case-Series Study

**DOI:** 10.1155/2018/5854710

**Published:** 2018-09-24

**Authors:** Bin Shi, Rong-rong Zhang, Ying Liang, Xin-hui Wang, Rui Lang, Ren-huan Yu

**Affiliations:** ^1^Graduate School of Beijing University of Chinese Medicine, Beijing, China; ^2^China Department of Nephrology, Xiyuan Hospital of China Academy of Chinese Medical Sciences, Beijing, China

## Abstract

**Background:**

The treatment of adult refractory idiopathic membranous nephropathy with steroid and other immunosuppressant-resistant nephrotic syndromes can be a significant challenge. We evaluated the efficacy and safety of the traditional Chinese medicine Jian Pi Qu Shi Formula (JPQSF) as a promising regimen.

**Methods:**

We analyzed 15 consecutive patients with biopsy-proven idiopathic membranous nephropathy who failed immunosuppressive therapy from October 2013 to January 2017. JPQSF was administered orally two times per day, respectively, in the morning and at night for 6 months. All patients had at least 1 year of follow-up. The primary endpoints included complete or partial remission. Secondary endpoints included change of clinical parameters and adverse events after 12 months of treatment.

**Results:**

After 12 months, complete remission was achieved in 13.3% of patients and partial remission in 66.7%, yielding a response rate of 80%. Proteinuria, serum albumin, and cholesterol were improved significantly (P<0.001, P<0.001, and P<0.05, respectively). After 1 year of treatment, proteinuria (mean ± SD) decreased from 5.93 ± 2.54 g per 24 h to 1.99 ± 1.17 g per 24 h (P<0.001). No serious adverse events occurred during the observation.

**Conclusions:**

JPQSF may be an alternative therapeutic option for steroid and general immunosuppressant-*resistant* membranous nephrotic syndrome patients, with a favorable safety profile. Larger and longer follow-up studies evaluating this regimen are warranted.

## 1. Introduction

Idiopathic membranous nephropathy (iMN), the major cause of nephrotic syndrome in Chinese adults [1], has dramatically increased in the last few years, especially in northern China [2]. Although, 30%–40% of iMN patients will undergo spontaneous and partial remission generally within 1 year from disease onset [3], approximately 40% of patients eventually develop end-stage renal disease within 10 years after diagnosis [4]. The 2012 Kidney Disease Improving Global Outcomes (KDIGO) guidelines recommend an initial immunosuppressive regimen that combines corticosteroids and an alkylating agent or calcineurin inhibitor (CNI) as the first-line choice in patients with persistent nephrotic syndrome. Such regimen can achieve relatively high remission rates and improve 10-year renal survival; however, still approximately 20% of iMN patients fail to achieve complete remission and have steroid-resistant nephrotic syndrome (SRNS), and 20%-30% of patients experience relapses after remission was achieved [5, 6]. In Asia, ~5-14% of refractory patients progress to end-stage renal disease (ESRD) [3, 4]. Refractory membranous nephropathy (RMN) is characterized by recurrence or resistance to therapy mentioned above. The treatment of RMN remains challenging. Patients experiencing long-term use of steroids and immunosuppressants can be associated with significant adverse effects such as nephrotoxicity and infection [7], which makes the treatment of RMN more difficult. Therefore, effective and safe therapeutic strategies for RMN need to be explored.

Traditional Chinese medicine (TCM) practitioners have been treating kidney diseases for several centuries in China and other countries in Asia and have accumulated extensive clinical experiences [8, 9]. TCM theoretical system takes physiology and pathology of* zang-fu* organs as its basis and differentiation of syndromes and treatment as its diagnostic and therapeutic features. The practice of traditional medicine is based on the theory of TCM whose basic premise is the idea of harmony. Through cumulative empirical experience of experts, patients with iMN have been treated with different formulations of herbs such as* Astragalus membranaceus* and* Angelica sinensis*, demonstrated to be effective on proteinuria in animal studies [10, 11]. Shenqi particle is a formulation that has been studied for the treatment of iMN in an open controlled clinical trial [12], but there has been no studies with a traditional Chinese medicine herbal formulation in patients with RMN.

Jian Pi Qu Shi Formula (JPQSF) has been developed for the treatment of iMN in Xiyuan Hospital, affiliated with China Academy of Chinese Medical Science. There are 10 herbs in JPQSF, which are selected based on the theory of traditional Chinese medicine and extensive experience from Professor Ren-huan Yu who has been practicing traditional Chinese medicine to treat patients with kidney disease over several decades. JPQSF is used to invigorate spleen and dispel dampness, thus restoring the harmony of Yin and Yang.

We here present a retrospective analysis of the outcomes of 15 consecutive RMN patients treated with JPQSF to examine the therapeutic efficacy of this regimen.

## 2. Methods

### 2.1. Study Setting

This was a retrospective case-series study, including all patients with histologically proven membranous nephropathy presenting to the Department of Nephropathy in Xiyuan Hospital.

### 2.2. Participant Selection

This study was conducted on patients with histologically proven membranous nephropathy presenting to the Department of Nephropathy in Xiyuan Hospital between October 2013 and January 2017; they were screened and selected based on following criteria: (1) already failed a regimen of cyclical monthly steroid and cyclophosphamide and/or CNI to induce a partial remission after at least six months of therapy; notably, all the patients have stopped such treatment before the study; (2) according to the syndrome of spleen deficiency and dampness excess; (3) medical records materials being completed. In accordance with “the guiding principles for the clinical study of new drugs for use in traditional Chinese medicine” released in 2002 and TCM theory of combination of disease and syndrome, combined with years of clinical observation and experience in our department, we summarized the standards of spleen deficiency and dampness excess syndrome in RMN, being formulated as follows: Primary symptoms are mental fatigue, soreness of waist, and edema. Secondary symptoms are (1) reduced appetite, insufficiency with chills, loose stools, or diarrhea and (2) dim complexion, frequency of urination, or frequency of urination at night. Tongue and pulse symptoms include (1) enlarged and tooth-marked tongue and greasy coating and (2) slow and sunken pulse. Participants can be diagnosed with spleen deficiency and dampness excess syndrome if they meet primary symptom and one of secondary symptoms and tongue or pulse. Patients were considered to have the nephrotic syndrome if they had a proteinuria*⩾*3.5 g per 1.73 m^2^ per 24 h, a serum albumin<3 g per L, and peripheral edema and are on optimal therapy with angiotensin converting enzyme inhibitors and/or angiotensin receptor blockers. Patients were excluded if they had any of the following conditions: (1) other types of membranous nephropathy, such as secondary membranous nephropathy, which were ruled out by obtaining patients' medical histories of malignancy, review of medications for potential drug use, contact with poison and by screening for laboratory tests, such as for hepatitis B and C viruses and antinuclear antibody testing; (2) diabetes or hemoglobin A1c level >6.2 mmol per L; (3) those who had received steroids or immunosuppressive drugs but with lack of standardization; (4) other types of syndromes in patients treated at Xiyuan Hospital, such as kidney Qi deficiency, Qi and Yin deficiency, stagnation of phlegm, and blood stasis. The present study was approved by the Ethics Committees of Xiyuan Hospital. In view of the retrospective nature of the study, a collection of informed consent was abandoned.

### 2.3. Experimental Treatment

**Figure 1:**
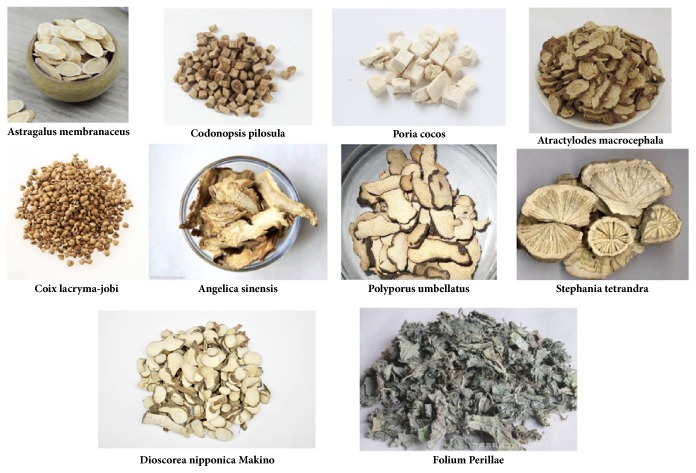
Pictures of medicinal slices in JPQSF.

Eligibility required a supportive treatment that was adjusted to ensure that they were receiving the maximum labeled or tolerated dose of RAS blockade, along with optimized blood pressure control according to 2012 KDIGO guidelines for glomerulonephritis and hypertension. Patients with syndrome of spleen deficiency and dampness excess were prescribed JPQSF. JPQSF is made of 10 different Chinese herbs (Table 1, Figure 1). It was prepared and supplied by the TCM Department of Xiyuan Hospital. Dosage of each herb in the formula changes according to the severity of symptoms. Three dosage forms, namely, decoction, concentration, and particle, were available for patients in the light of their requirements. JPQSF was administered orally two times a day, respectively, in the morning and at night for 6 months.

### 2.4. Data Collection

All patients were followed up at regular intervals at least one year. Patients were scheduled to have study visits at baseline and months 3, 6 and 12. Additional telephone or face-to-face (at the choice of the investigator) visits were required if patients were not available. At each study visit, clinical and laboratory parameters including 24 hour urinary protein, serum albumin, plasma creatinine, liver enzymes (alanine and aspartate aminotransferase), and blood cell counts were measured.

### 2.5. Measures

#### 2.5.1. The Primary Outcome

The primary outcome was attainment of remission rate, including complete and partial remissions. Remission was defined according to 2012 KDIGO guidelines as follows: (1) complete remission (CR) was defined as a reduction in proteinuria to protein excretion 0.3 g per 24 h. (2) Partial remission (PR) was defined as the case of urinary protein excretion < 3.5 g per 24 h with* ⩾*50% reduction compared with baseline. Any patient reaching a CR or PR was considered a treatment success.

#### 2.5.2. The Secondary Outcome

The secondary outcomes included the change in proteinuria, serum albumin level, evaluated glomerular filtration rate (eGFR), doubling of serum creatinine level, end-stage renal disease, and death. Because albumin level may be an earlier marker of response than end points defined by proteinuria only [13], we also considered a post hoc composite end point defined as reduction of proteinuria > 50% and increase of serum albumin level > 30% at month 6 and 12 of follow-up.

#### 2.5.3. Adverse Events

Any symptoms and adverse effects such as infections, hospitalizations, or other complications were recorded at each follow-up visit or immediately when this happened. Serious adverse events (SAEs) were defined according to the International Conference on Harmonization Guideline for Clinical Safety Data Management.

### 2.6. Data Analysis

Baseline characteristics of the study population were presented as frequencies and percentages for qualitative variables and medians and interquartile ranges (IQR) for continuous variables. Remission rates were expressed as frequencies or percentages and their 95% confidence intervals (95% CIs). For the comparison between two groups among the three groups, Bonferroni's correction of the Mann-Whitney test was used. Statistical analyses were carried out using SPSS, version 22. All P values were 2-sided, and P values less than 0.05 were considered statistically significant.

## 3. Results

From October 2013 to January 2017, 15 consecutive patients with RMN receiving JPQSF were enrolled. All the patients received maximal tolerable doses of angiotensin receptor blockers (systolic blood pressure achieved was <130 mmHg in all the subjects) and atorvastatin.

There were 5 female and 10 male patients, with an average age of 47.6 ± 8.4 years. All the subjects had been previously administered and failed at least six-month course of immunosuppressant treatment: 6 patients resistant to corticosteroid plus cyclophosphamide, cyclosporine in 7 patients with relapsing disease, and 2 patients intolerant to tacrolimus treatment (Table 2).

At entry, the mean level of proteinuria was 5.93 ± 2.54 g per 24 h (median 5.10, range 3.10-13.00). Serum albumin was 23.88 ± 3.28 g per L. Serum creatinine was 80.33 ± 26.42 umol per L and the mean creatinine clearance was 96.48 ± 28.91 ml per min per 1.73 m^2^ (Table 2). All the study subjects had nephrotic range proteinuria (>3.5 g per d), but for one who had subnephrotic proteinuria (3.1 g per 24 h) and was treated as he had anasarca with severe hypoalbuminemia (27.1 g per L) (Table 3).

**Figure 2:**
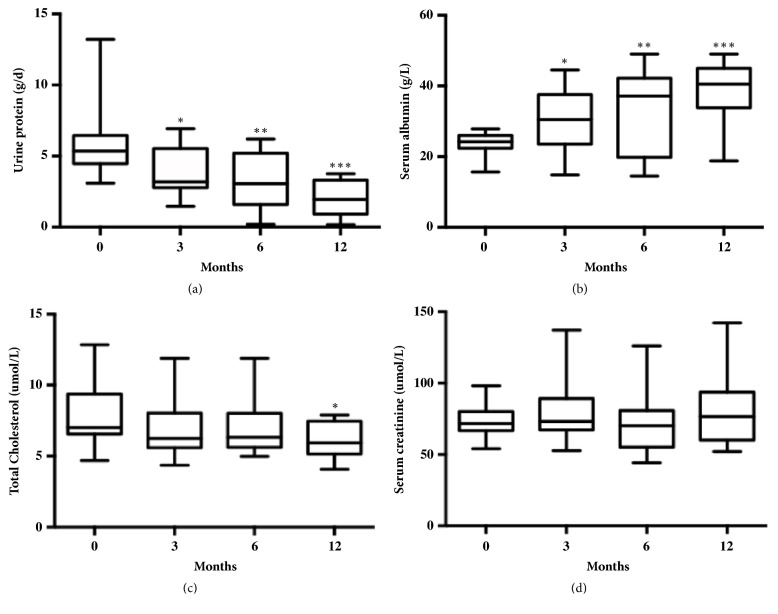
Repeated measurements of selected laboratory parameters from JPQSF administration (baseline) to study end (month 12). A repeated-measures ANOVA examining the changes of proteinuria, serum albumin, and cholesterol from baseline to month 12 showed significant time effects (P< 0.05). (a) Proteinuria: ∗ indicates Bonferroni correction of the baseline and 3 months after treatment values, P<0.05; ∗∗ indicates Bonferroni correction of the baseline and 6 months after treatment values, P<0.05; ∗∗∗ indicates Bonferroni correction of the baseline and 12 months after treatment values, P<0.001. (b) Serum albumin: ∗ indicates Bonferroni correction of the baseline and 3 months after treatment values, P<0.05; ∗∗ indicates Bonferroni correction of the baseline and 6 months after treatment values, P<0.05; ∗∗∗ indicates Bonferroni correction of the baseline and 12 months after treatment values, P<0.001. (c) Cholesterol: ∗ indicates Bonferroni correction of the baseline and 12 months after treatment values, P<0.05; (d) Serum creatinine: baseline value and values during treatments compared with baseline values before treatment; serum creatinine does not show significant changes at the end of 12 months (P>0.05).

In the 15 patients, being treated with JPQSF for 6 months, the remission rate was 66.7%; the CR was achieved in one patient and PR in nine patients (Table 5), and proteinuria decreased from 5.93 ± 2.54 g per 24 h to 3.01 ± 2.02 g per 24 h at 6 months (P<0.05, Figure 2(a), Table 4). All the patients completed 6 months of follow-up, and proteinuria decreased from 5.93 ± 2.54 g per 24 h to 1.99 ± 1.17 g per 24 h at 12 months (P<0.001, Figure 2(a) and Table 4). 12 months later, the remission rate was 80%, and CR was achieved in two patients and PR in ten patients (Table 5). It meant that the longer follow-up time, the more remission we may observe. In the 3 patients who did not respond, proteinuria at 12 months was not significantly different from baseline (Table 4).

The reduction in proteinuria was paralleled by a progressive and significant increase in serum albumin levels from 23.88 ± 3.28 g per L at baseline to 38.96 ± 9.08 g per L at 12 months (P <0.001, Figure 2(b), Table 4). Total serum cholesterol levels also decreased by 12 months (P<0.05, Figure 2(c)). There were no significant changes in 24 h creatinine clearances obtained at baseline versus at 12 months (P >0.05, Figure 2(d)). However, in the two patients who had the lowest creatinine clearance at entry (57.72 and 41.34 ml per min per 1.73 m^2^), renal function was improved and stable (68.84 and 42.17 ml per min per 1.73 m^2^).

Over six months of follow-up, there was no report of any adverse events.

## 4. Discussion

Since then, information about treatment of iMN resistant to standard immunosuppressive therapy or frequently relapsing in adults remains a therapeutic challenge for nephrologists. Several clinical trials that have been conducted to evaluate new strategies in managing these patients have emerged recently to evaluate the efficacy of different therapy regimens. Cyclosporine (CyA) has been used as an effective agent for iMN with SRNS [14, 15] and its combination with steroids is recommended in some guidelines for SRNS therapy. A cohort study has reported a benefit of the combination of MZR and steroids treatment in 14 of 22 adult patients with steroid-resistant iMN in Japan [16]. Wei Chen [17] et al. conducted a multicenter prospective study evaluating TAC plus prednisone regimen for adult Chinese patients with refractory membranous nephrotic syndrome, which showed a favorable effect on the outcome of patients. The available data on rituximab treatment in RMN describes different regimens of B cell depletion [18]. Other regimen of rituximab and plasmapheresis for severe therapy-refractory iNM in adults was published by Ming Wen et al. [19]. In addition, a case report [20] showed that using an icodextrin-single Peritoneal dialysis (PD) therapy for RMN alleviated water stagnation while preserving residual renal function.

However, these therapies do not always induce remission and also cause significant adverse effects; regimen like rituximab or tacrolimus may modulate immunity and increase susceptibility to infection like Pneumocystis jiroveci pneumonia [21] and other side effects including nephrotoxicity and hypertension. Therefore, in China and other Asian countries, many patients with RMN search for an alternative therapy, such as TCM.

TCM practitioners have been treating kidney diseases for several centuries in China and other countries in Asia and have accumulated extensive clinical experiences [22]. The overarching principle in the practice of TCM is the focus on individual assessment and treatment to coordinate the natural balance of the Yin and Yang, which are two major opposing forces of the body represented in the ancient Chinese Taoism philosophy. In the practice of TCM, it is generally considered that multiple herbal medications are more effective than a single herbal agent. Therefore, prescriptions of TCM usually combine several types of herbs or minerals, where one herb represents the principal component and others serve as adjunctive agents, assisting the effects or facilitating the delivery of the principal component.

With recent integration of TCM and western medicine, diagnosis of CKD by practitioners of TCM has supplemented traditional diagnostic approach with western molecular and imaging diagnostic tools. The current treatment of CKD in TCM is often achieved by combining TCM and western pharmacologic agents. The therapeutic principles of TCM for CKD include ‘replenishing vital energy and nourishing blood,' ‘clearing heat and eliminating dampness,' and ‘coordinating Yin and Yang in the body' [23]. Hundreds of herbs used in prescriptions of a single herb, decoctions of multiple herbs, or patent medicines have been used to treat patients with CKD. These prescriptions have effects including promotion of diuresis, reduction of proteinuria, and improvement of renal function [24].

Although many small clinical studies published in Chinese journals support a kidney protective effect of traditional medicine for CKD, large and well-designed randomized controlled trials (RCTs) are still lacking. However, progress has been made recently in this field. Large randomized clinical trials of TCM in kidney disease include Shenqi particle and Huangkui capsule. To our knowledge, Shenqi particle [12] was the first open-label, multicenter, randomized, controlled trial in the field of traditional medicines in kidney disease of idiopathic membranous nephropathy. Results showed that reduction of urinary protein excretion was similar in the Shenqi particle and control groups. However, eGFRs declined less in the Shenqi particle group than in controls. In addition, patients in the Shenqi particle group had fewer side effects than those in the standard therapy group. These findings suggested that Shenqi particle could be an alternative therapy for patients with idiopathic membranous nephropathy. Another published RCT assessed the efficacy and safety of* Abelmoschus manihot (L)* medic (AM) [25] in patients with primary glomerular disease. Results showed that patients treated with AM or AM plus losartan had significantly less proteinuria than those treated with losartan alone. Small clinical trials of TCM in kidney disease include* Tripterygium wilfordii Hook F *(TWHF),* triptolide*. In an uncontrolled trial, Chen et al. [26] examined the effects of TWHF in 9 patients with polycystic kidney disease and proteinuria. They found that 6 months of treatment with TWHF reduced proteinuria, preserved kidney function, and diminished total kidney volume in these patients, suggesting multiple effects of TWHF. Another study looked at the clinical efficacy of* triptolide* in children with moderately severe Henoch-Schönlein purpura concluded that* triptolide* is effective in relieving short-term symptoms.

Recently, several systematic reviews and meta-analyses were performed on RCTs using different* Tripterygium* preparations, TWHF,* Astragalus*,* Rheum officinale *(a type of rhubarb), standard care, or other immunosuppressive treatment in patients with CKD. On account of the small sample size, short follow-up, and concerns for methodologic bias, the quality of evidence was suboptimal, and there is insufficient evidence to conclude that such TCM regimens are as effective as other regimens. Therefore, large RCTs are required to confirm these findings.

Mechanisms of action have been studied for some herbs. Their effects are mainly related to anti-inflammation, antioxidation, antifibrosis, regulation of immune system, anticoagulation, and improvement of metabolic disturbance [27]. Active ingredients were purified from herbs that have been studied in CKD. Many traditional medicines have been shown to have both anti-inflammatory and antioxidative effects, such as* Astragalus membranaceus*, TWHF,* Saikosaponin A*,* radix puerariae *(kudzu root), AM, and* Cortex moutan *(root bark of* Paeonia suffruticosa*) both in vitro and in vivo trials. Some traditional medicines like* Astragalus*, TWHF,* Saikosaponins*, and* Rhein *(an acidic compound derived from* rhubarb*) have also been reported to have antifibrotic effects and to inhibit mesangial cell proliferation and matrix synthesis. In addition, several traditional medicines such as* Astragalus*,* Triptolide*,* Saikosaponins*, and* Emodin *are known to regulate the immune system [9]. Therefore, these traditional medicines have been used as alternatives to immunosuppressive medications for the treatment of primary glomerular disease.

Owing to the rapid economic growth and scientific development over the past decade, the Chinese government has supported studies to examine the scientific basis of TCM using advanced cell and molecular biology approaches. A rapid development of TCM is expected over the next decade. Nowadays more and more animal studies have confirmed the biological activity and therapeutic effects of several traditional Chinese herb medicines in CKD.

In the present study, the traditional Chinese medicine formulation of JPQS decoction was effective at inducing remission in patients with RMN. In addition, JPQSF had a safety profile.

JPQSF contains 10 different herbs and the exact active components for the antiproteinuric effect are unclear. However, the therapeutic activity of JPQSF could be related to the herbal component* Astragalus*.* Astragalus* has been shown to have strong anti-inflammatory and antioxidative stress effects in different cell types, including kidney cells, through suppression of p38 MAPK (mitogen-activated protein kinase), NF-*κ*B (nuclear factor *κ*B), and Toll-like receptor–mediated pathways [28, 29].* Astragalus* has been shown to reduce proteinuria and attenuate kidney injury in several animal models of kidney disease [9]. In several small clinical trials, injection of* Astragalus* extract in patients with different forms of chronic glomerulonephritis has been shown to reduce proteinuria [17, 18] and ameliorate dyslipidemia [19].* Dioscoreae Nipponicae Rhizoma *has shown potent anti-inflammatory and antiarthritic effects in vitro and in vivo studies [30]. It has been reported to attenuate rheumatoid arthritis by regulating the expression of iNOS, COX-2, and NF-*κ*B expression. The role of other herbs in renal protection requires further study.

In the present study, cases with severe adverse events were not observed. The safety profile of JPQSF suggests an important advantage of using TCM in RMN patients and/or elderly patients with other severe illness because these patients are more susceptible to the adverse effects of immunosuppressive agent.

One of the findings in this study was that a significant increase in serum albumin level before improvement in proteinuria was observed, which suggests that the mechanism underlying JPQSF's initial effect on plasma albumin level might be independent of the reduced urinary protein excretion.* Astragalus* and* Angelica*, two components of JPQSF, have been shown to improve protein metabolic disorder in patients with nephrotic syndrome by promoting net protein synthesis. These studies suggest that JPQSF may improve serum albumin levels through these mechanisms.

Our study has several important limitations. The major limitation of this study is that we were not able to follow up these patients for a longer period to observe the complete remission rate and relapsing rate because these patients are from different areas of China. Another limitation is that the exact active components of JPQSF and mechanisms of action remain unclear. This study is retrospective single-center, of limited size, and lacks a comparator group. Statistical results must be interpreted with caution in this setting. Larger studies are required to confirm our findings.

## 5. Conclusion

In summary, JPQSF was effective and safe for the treatment of RMN and may be an alternative therapeutic option for the treatment of refractory nephrotic iMN patients who failed to respond to steroid and other conventional immunosuppressant agents. Over a follow-up time of 1 year, 80% of patients achieved remission. Future randomized clinical trials with long-term follow-up are inevitably needed to establish JPQSF as evidence-based treatment option in this part of refractory nephrotic syndrome.

## Figures and Tables

**Table 1:** Components of JPQSF.

**Latin Binomial Name**	**English Name**	**Part Used**	**Origin of Product** ^**a**^	**Type of Product**	**Weight (g)**
*Astragalus membranaceus *	Radix Astragali	Root	Gansu	Raw (dry)	30
*Codonopsis pilosula*	Radix Codonopsitis Pilosulae	Root	Gansu	Raw (dry	15
*Poria cocos *	Sclerotium Poriae Cocos	Sclerotium	Anhui	Raw (dry	30
*Atractylodes macrocephala *	Rhizoma Atractylodis Macrocephalae	Stem	Zhejiang	Raw (dry	20
*Angelica sinensis*	Radix Angelicae sinensis	Root	Gansu	Raw (dry)	15
*Coix lacryma-jobi *	Semen coicis	Seed	Guizhou	Raw (dry)	30
*Polyporus umbellatus *	Sclerotium polypori Umbrellati	Sclerotium	Shanxi	Raw (dry)	15
*Stephania tetrandra*	Radix Stephaniae Tetrandrae	Root	Zhejiang	Raw (dry)	20
*Dioscorea nipponica Makino*	Dioscoreae Nipponicae Rhizoma	Root	Liaoning	Raw (dry)	30
*Folium Perillae*	Perillae Folium	Foliage	Shanxi	Raw (dry)	15

__^a^Province.

**Table 2:** Baseline characteristics of the RMN patients receiving JPQSF regimen.

**Characteristics**	**Value(n=15)**
Age, mean (SD), y	47.6(8.4)
Female, no. (%)	5(33.3)
Urine protein excretion, mean (SD), g/24 h	5.93(2.54)
Serum albumin, mean (SD), g/L	23.88(3.28)
Estimated GFR, mean (SD), ml/min/1.73 m^2^	96.48(28.91)
Serum creatinine, mean (SD), umol/L	80.33(26.42)
Total Cholesterol, mean (SD), mol/L	7.82(2.23)
Triglycerides, median (IQR), mol/L	3.10(1.53)
Histology stageb no. (%)	
I	5(33.3)
II	7(46.7)
I-II	3(20.0)
Previous treatment no. (%)	
Prednisone + Cyclophosphamide	6(40.0)
Prednisone +Cyclosporine	7(46.7)
Tacrolimus	2(13.3)

Abbreviations. GFR: glomerular filtration rate.

__^a^Estimated with the use of the Chronic Kidney Disease Epidemiology Collaboration creatinine equation.

**Table 3:** Main clinical and laboratory characteristics at study entry (baseline) of individual patients with RMN.

**Patient number**	**1**	**2**	**3**	**4**	**5**	**6**	**7**	**8**	**9**	**10**	**11**	**12**	**13**	**14**	**15**
**Age (years) **	47	62	44	49	45	26	40	52	17	36	28	43	54	57	61
**Gender (male/female) **	M	F	M	F	F	M	M	M	M	M	F	F	M	M	M
**Histology stage (I-IV)**	I	II	II	I-II	II	II	I	II	I-II	I	II	II	I-II	I	I
**Urinary protein excretion (g per 24 h)**	4.4	5.6	4.5	4.0	7.3	4.8	5.7	10.0	6.2	3.1	5.1	6.1	5.1	4.2	13.0
**Serum albumin(g per L)**	27.9	22.5	24.2	25.3	22.1	23.7	25.9	19.0	24.0	27.1	24.5	26.3	24.2	23.7	15.8
**Serum creatinine (umol per L)**	82.0	91.9	57.0	52.0	97.0	75.6	93.0	82.0	49.2	79.0	65.0	59.0	84.0	86.0	154.0
**Creatinine clearance (ml per min per 1.73 m** ^**2**^ **) **	97.7	57.7	118.7	108.1	60.9	119.9	88.2	90.3	152.4	109.8	111.5	108.2	90.4	85.9	41.3

**Table 4:** Time course of urinary protein excretion (g per 24 h) in individual patients with RMN from entry into the study (baseline) to end of study (month 12).

**Patient no.**	**Primary IST**	**Indication**	**Baseline**		**Month 3**		**Month 6**		**Month 12**		**Baseline to month 12** ^**a**^	
			**Urinary protein excretion**	**Serum albumin**	**urinary protein excretion**	**Serum albumin**	**urinary protein excretion**	**Serum albumin**	**urinary protein excretion**	**Serum albumin**	**urinary protein excretion**	**Serum albumin**
1	cCTX/GC	Resistant	4.40	27.90	6.31	20.20	6.18	20.20	3.59	35.20	-0.81	7.30
2	CsA/GC	Intolerant	5.60	22.50	2.80	26.70	5.70	18.00	3.75	18.80	-1.85	-3.70
3	CsA/GC	Relapse	4.47	24.20	2.82	37.90	1.60	40.00	1.13	41.00	-3.34	16.80
4	cCTX/GC	Resistant	4.00	25.30	2.65	37.50	0.56	46.10	1.28	47.00	-2.72	21.70
5	cCTX/GC	Resistant	7.28	22.10	5.07	24.66	2.87	32.89	1.33	45.00	-5.95	22.90
6	TAC	Intolerant	4.80	23.70	3.08	29.30	1.16	44.60	1.60	44.32	-3.20	20.62
7	TAC	Intolerant	5.72	25.92	3.18	34.54	3.78	34.97	2.78	40.00	-2.94	14.08
8	CsA/GC	Relapse	10.00	19.00	6.92	25.40	3.34	28.10	2.11	38.60	-7.89	19.60
9	cCTX/GC	Resistant	6.17	24.00	4.58	30.40	1.63	46.60	0.17	47.36	-6.00	23.36
10	CsA/GC	Relapse	3.10	27.10	1.45	43.00	0.23	49.00	0.21	49.00	-2.89	21.90
11	CsA/GC	Relapse	5.10	24.50	2.00	44.50	1.80	41.40	1.13	45.00	-3.97	20.50
12	cCTX/GC	Resistant	6.08	26.30	3.44	30.60	3.25	39.40	1.78	42.00	-4.30	15.70
13	CsA/GC	Resistant	5.08	24.20	2.47	41.80	1.58	39.70	2.45	39.70	-2.63	15.50
14	cCTX/GC	Relapse	4.20	23.70	6.80	14.80	6.20	14.50	3.30	21.80	-0.90	-1.90
15	CsA/GC	Resistant	13.00	15.80	5.53	15.40	5.20	18.00	3.32	29.63	-9.68	13.83
Mean	/	/	5.93	23.88	3.94	30.45	3.01	34.23	1.99	38.96	-3.94	15.21
SD	/	/	2.54	3.28	1.78	9.47	2.02	11.69	1.17	9.08	2.48	8.48
Median	/	/	5.10	24.20	3.17	30.40	2.86	39.40	1.78	41.00	-3.20	16.80

Abbreviations. RMN: refectory membranous nephropathy; IST: immunosuppressive therapy; cCTX/GC: cyclical cyclophosphamide and steroids; TAC: tacrolimus; CsA/GC: cyclosporin and steroids.

^a^Paired t-test P<0.001 for 12-month change.

**Table 5:** Effects of JPQSF on prespecified primary outcomes.

	**Month 6**	**Month 12**	**P**
Number	15	15	
CR	1 (6.7%)	2 (13.3%)	0.598^a^
PR	9 (60%)	10 (66.7%)	0.705^a^
CR+PR	10 (66.7%)	12 (80.0%)	0.682^a^

Abbreviations. CR: complete remission; PR: partial remission.

^a^Statistics were performed by Fisher exact test due to low frequency.

## Data Availability

The data used to support the findings of this study are available from the corresponding author upon request.
